# Supplementary material for: Context-specific emergence and growth of the SARS-CoV-2 Delta variant
Source: Nature. 2022 Aug 11;610(7930):154–60. doi: 10.1038/s41586-022-05200-3 (PMC9534748; doi:10.1038/s41586-022-05200-3)
Supplement: Supplementary file 2 — Reporting Summary [file 41586_2022_5200_MOESM2_ESM.pdf]

Corresponding author(s): Pybus, Kraemer, Rambaut

Last updated by author(s): 21/12/2021

Reporting Summary

Nature Portfolio wishes to improve the reproducibility of the work that we publish. This form provides structure for consistency and transparency in reporting. For further information on Nature Portfolio policies, see our Editorial Policies and the Editorial Policy Checklist.

Statistics

For all statistical analyses, confirm that the following items are present in the figure legend, table legend, main text, or Methods section.

n/a	Confirmed	
☐	☒	The exact sample size (*n*) for each experimental group/condition, given as a discrete number and unit of measurement
☒	☐	A statement on whether measurements were taken from distinct samples or whether the same sample was measured repeatedly
☐	☒	The statistical test(s) used AND whether they are one- or two-sided<br>*Only common tests should be described solely by name; describe more complex techniques in the Methods section.*
☐	☒	A description of all covariates tested
☒	☐	A description of any assumptions or corrections, such as tests of normality and adjustment for multiple comparisons
☐	☒	A full description of the statistical parameters including central tendency (e.g. means) or other basic estimates (e.g. regression coefficient) AND variation (e.g. standard deviation) or associated estimates of uncertainty (e.g. confidence intervals)
☐	☒	For null hypothesis testing, the test statistic (e.g. *F*, *t*, *r*) with confidence intervals, effect sizes, degrees of freedom and *P* value noted<br>*Give P values as exact values whenever suitable.*
☐	☒	For Bayesian analysis, information on the choice of priors and Markov chain Monte Carlo settings
☐	☒	For hierarchical and complex designs, identification of the appropriate level for tests and full reporting of outcomes
☐	☒	Estimates of effect sizes (e.g. Cohen's *d*, Pearson's *r*), indicating how they were calculated

Our web collection on statistics for biologists contains articles on many of the points above.

Software and code

Policy information about availability of computer code

Data collection	*Provide a description of all commercial, open source and custom code used to collect the data in this study, specifying the version used OR state that no software was used.*
Data analysis	Code to partially reproduce the statistical analyses on Delta growth can be found here: https://github.com/sumalibajaj/Delta-Statistical-analysis-share. The code and accession ids of sequences used to run the phylogenetic analysis as well as an GISAID acknowledgment table are available here: https://github.com/COG-UK/Delta-analysis.<br>Explicit details about the analyses and their dependencies can be found in the repositories listed above. Details regarding COGUK genomic processing pipeline can be found at https://github.com/COG-UK/datapipe. In particular these analyses use the following software.<br>minimap2v2.17<br>FastTreev2.1.10<br>BEASTv1.10.4 and BEASTv1.10.4 (commit:d1a45)<br>Tracer v1.7

For manuscripts utilizing custom algorithms or software that are central to the research but not yet described in published literature, software must be made available to editors and reviewers. We strongly encourage code deposition in a community repository (e.g. GitHub). See the Nature Portfolio guidelines for submitting code & software for further information.

Data

Policy information about availability of data

All manuscripts must include a data availability statement. This statement should provide the following information, where applicable:

- Accession codes, unique identifiers, or web links for publicly available datasets
- A description of any restrictions on data availability
- For clinical datasets or third party data, please ensure that the statement adheres to our policy

UK genome sequences used were generated by the COVID-19 Genomics UK consortium (COG-UK, https://www.cogconsortium.uk/). Data linking COG-IDs to location have been removed to protect privacy, however if you require this data please visit https://www.cogconsortium.uk/contact/ for information on accessing consortium-only data. The Google COVID-19 Aggregated Mobility Research Dataset used for this study is available with permission from Google LLC. Code to reproduce the statistical analyses on Delta growth can be found here: https://github.com/sumalibajaj/Delta-Statistical-analysis-share. The code and accession ids of sequences used to run the phylogenetic analysis as well as an GISAID acknowledgment table are available here: https://github.com/COG-UK/Delta-analysis.

Field-specific reporting

Please select the one below that is the best fit for your research. If you are not sure, read the appropriate sections before making your selection.

☐ Life sciences ☐ Behavioural & social sciences ☒ Ecological, evolutionary & environmental sciences

For a reference copy of the document with all sections, see nature.com/documents/nr-reporting-summary-flat.pdf

Ecological, evolutionary & environmental sciences study design

All studies must disclose on these points even when the disclosure is negative.

Study description	Our study characterizes the importation and subsequent spread of the SARS-CoV-2 Delta variant of concern in England. We use the UK genomic data produced by COG-UK to highlight the temporal and spatial dynamics of imported transmission lineages, and novel epidemiological models to determine factors that led to the growth of Delta across both the UK and US.
Research sample	The phylogenetic analysis used UK sequences produced by COG-UK and international sequences shared on GISAID. The UK data set consists of pillar 2 English samples taken as part of the national testing infrastructure for community surveillance. These samples were chosen to as they best represent a random sample of lineages circulating in the general English public at the time of study. Only samples identified as the Delta variant of concern were included in this study. UK genome sequences used were generated by the COVID-19 Genomics UK consortium (COG-UK, https://www.cogconsortium.uk/)
Sampling strategy	No subsampling strategy was used in this study. All sequences identified as Delta were included. This resulted in a dataset of over 90,000 sequences. This is the largest phylogenetic analysis of it's kind, and used all available pillar 2 English samples generated by COGUK. This was done to generate the best approximation of circulating lineages in England at the time of study.The density of the data allows for detailed modeling of geographic spread in England.
Data collection	English samples were taken and sequenced by COG-UK affiliated partners across the UK and shared according to the COG-UK data sharing agreement. Post-processing of genomic data was done as part of the daily COG-UK 'datapipe/phylopipe' hosted on CLIMB. Four sources of data were compiled to provide the travel history for laboratory confirmed cases, depending on availability for each individual case: (1) public health passenger locator forms are required for entry into the UK; (2) routine public health contact tracing data including UK Health Security Agency Second Generation Surveillance System (SGSS)89, (3) COVID-19 test requests with reported travel associations and (4) responses to additional telephone interviews for cases. This data was processed by authors in PHE and shared with others under the appropriate data sharing agreement. We used two human mobility datasets, one a country flight dataset from IATA and another from the Google Mobility Research Dataset which was aggregated to the country level and UTLA level in the UK and state level in the USA.
Timing and spatial scale	All SARS-CoV-2 non-UK genomes in GISAID and pillar 2 samples from England that were identified as Delta up until June 15, 2021 were included in this study. The spatial resolution of the global analyses were at the country level and for the detailed phylogeographic analyses at the postcode level.
Data exclusions	Sequences with known quality flaws as well as those that failed to pass standard quality measures (e.g. those with low sequence coverage and temporal outliers) were removed from the study.
Reproducibility	This study is based on epidemiological data and subsequent modeling. All code to reproduce the results and what data we are able to share has been shared to make reproducing such analysis in this and other settings possible.
Randomization	Randomization was not relevant in this study, as it was based on epidemiological observations in the general public and there were no experimental treatments applied.
Blinding	Blinding was not relevant to this study design,as it was based on epidemiological observations in the general public and there were no experimental treatments applied.

Did the study involve field work? ☐ Yes ☒ No

Reporting for specific materials, systems and methods

We require information from authors about some types of materials, experimental systems and methods used in many studies. Here, indicate whether each material, system or method listed is relevant to your study. If you are not sure if a list item applies to your research, read the appropriate section before selecting a response.

Materials & experimental systems

n/a	Involved in the study
☒	☐ Antibodies
☒	☐ Eukaryotic cell lines
☒	☐ Palaeontology and archaeology
☒	☐ Animals and other organisms
☒	☐ Human research participants
☒	☐ Clinical data
☒	☐ Dual use research of concern

Methods

n/a	Involved in the study
☒	☐ ChIP-seq
☒	☐ Flow cytometry
☒	☐ MRI-based neuroimaging
